# Injectable conductive hydrogel electrodes for minimally invasive neural interfaces†

**DOI:** 10.1039/d4tb00679h

**Published:** 2024-07-30

**Authors:** Ines Kusen, Aaron Lee, Estelle A. Cuttaz, Zachary K. Bailey, Joshua Killilea, Shirine Merlo-Nikpay Aslie, Josef A. Goding, Rylie A. Green

**Affiliations:** a Department of Bioengineering, Imperial College London London SW7 2BX UK rylie.green@imperial.ac.uk; b Faculty of Medicine, Imperial College London London SW7 2BX UK

## Abstract

Soft bioelectronic neural interfaces have great potential as mechanically favourable alternatives to implantable metal electrodes. In this pursuit, conductive hydrogels (CHs) are particularly viable, combining tissue compliance with the required electrochemical characteristics. Physically-aggregated CHs offer an additional advantage by their facile synthesis into injectable systems, enabling minimally invasive implantation, though they can be impeded by a lack of control over their particle size and packing. Guided by these principles, an injectable PEDOT:PSS/acetic acid-based hydrogel is presented herein whose mechanical and electrochemical properties are independently tuneable by modifying the relative acetic acid composition. The fabrication process further benefits from employing batch emulsion to decrease particle sizes and facilitate tighter packing. The resulting material is stable and anatomically compact upon injection both in tissue phantom and *ex vivo*, while retaining favourable electrochemical properties in both contexts. Biphasic current stimulation yielding voltage transients well below the charge injection limit as well as the gel's non-cytotoxicity further underscore its potential for safe and effective neural interfacing applications.

## Abstract 2

An injectable PEDOT:PSS/acetic acid-based hydrogel was formulated with mechanical and electrochemical properties being independently tuneable. The material was characterised *in vitro* and *ex vivo*, with prospective applications in neural interfacing.

## Graphical abstract

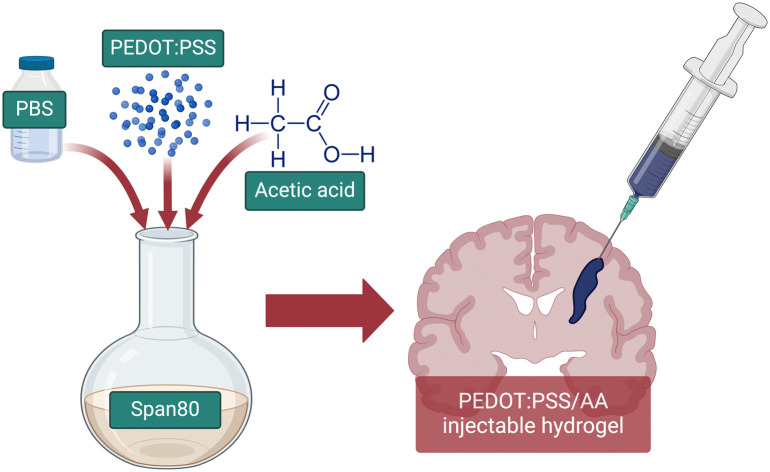

## Introduction

Implantable bioelectronic neural interfaces are situated at the forefront of medical device technology while also promising an unparalleled understanding of the brain.^1^ Clinically, implanted neural electrodes have remained largely reliant on stiff and inert metals like platinum (Pt) and iridium (Ir) due to their excellent electrical conductivity.^2^ However, their mechanical incompatibility with soft neural tissue, poor charge transfer characteristics, and highly invasive implantation surgeries have necessitated the development of neural interfaces with improved bio-integration.^3–5^ Although thin-film mesh electronics have been proposed as an injectable solution for reducing scar tissue formation and infection risk, they remain sensitive to substrate failure and interconnect conductivity.^6^ In contrast, injectable conductive hydrogels (CHs) have gained traction as hybrid materials that can be designed using concepts of tissue engineering to provide cell-supportive and functionally addressable environments for minimally invasive neural interfaces.^7^

Conductive hydrogels are often composed of a hydrophilic polymer and an electrically conductive component such as carbon nanotubes, metal particles and intrinsically conductive polymers.^8,9^ Although many composites have been developed as cell scaffolds or soft electronics, they are unsuitable for syringe injection due to the covalent gelation through chemical crosslinking.^10,11^ Non-covalent interactions and supramolecular gel assemblies offer an alternative pathway by supporting self-healing behaviour that ensures key properties can be retained following material delivery.^12^ An ideal injectable conductive hydrogel would need to be able to be manually extruded through a needle, possess shear-thinning behaviour, achieve sufficient percolation for electrical conductivity and be capable of effective charge transfer to tissue. Granular hydrogels have attracted attention for extrusion-based biofabrication as modular and highly tuneable systems that reflect many of the desired traits for injection.^12–14^ Recent work by the Bao group have demonstrated how a granular hydrogel system can be formulated by screening electrostatic interactions in aqueous dispersions of poly(3,4-ethylenedioxythiophene):polystyrene sulfonate (PEDOT:PSS) colloids to induce aggregation. Application of shear forces results in relative displacement between particles which can subsequently re-establish interparticle interactions and jam at zero shear while retaining electrical conductivity.

For continuous interfacing and tissue contact across a wide range of length scales, smaller particles offer improved tissue penetration and surface area for effective charge transfer.^13,15,16^ Batch emulsion has been shown to produce a range of droplet sizes that could facilitate particle size reduction in granular hydrogel systems.^14^ Moreover, particle size distributions produced by emulsions exhibit significant polydispersity due to the limited control over their formation.^17^ This especially presents an advantage when working with spherical particles since a large range of sizes minimises the void-space between them wherein the smaller particles are able to fill the spaces between the larger ones, thus improving local packing.^18,19^ Importantly, it has been shown that acid treatment of PEDOT:PSS materials can be used to enhance electrical conductivity, thus further contributing towards its effective charge transfer.^12,20–22^ By subsequently using acetic acid to support recovery of electronic conductivity, the resultant impact of the formulation on both flow behaviour and electrochemical response can be assessed.

Herein, we present an injectable, conductive hydrogel system composed of jammed PEDOT:PSS particles whose mechanical and electrical properties have been shown to be independently tailorable. Batch emulsion was employed to reduce aggregate size, thereby facilitating inter-particle packing while maintaining favourable charge transfer. Injection properties could be tuned by modifying the relative acetic acid composition without impeding electrochemical performance. Following injection into *ex vivo* tissue, the electrodes remain anatomically localised and, along with their retained functionality, offer new perspectives in minimally invasive neural interfacing.

## Methods

### Materials

PEDOT:PSS (dry re-dispersible pellets), acetic acid (ACS Reagent, ≥99.7%), Tween20, Span80, light mineral oil, phosphate-buffered saline tablets, agarose (powder, made as 0.6% w/v in 100 mL phosphate-buffered saline, PBS), and Lugol staining solution were purchased from Sigma-Aldrich. PBS was made by diluting tablets in deionised (DI) water (resistivity of 18.2 MΩ). Cell medium was composed of Dulbecco's Modified Eagle's Medium (DMEM) supplemented with 10% v/v foetal bovine serum (FBS) and 1% penicillin/streptomycin (P/S). All medium components were purchased from Thermo-Scientific. Rat Schwann cells (SCL 4.1/F7) were obtained from Sigma-Aldrich and maintained in incubators at 37 °C, 5% pCO_2_ and 100% humidity. Cytotoxicity was performed *via* staining with calcein-AM/ethidium homodimer-1 (live/dead assay, invitrogen). For coating the wire used in electrochemical characterisation, polyurethane (Pellethane PU80AE, Lubrizol), *N*,*N*′-dimethylacetamide, and lithium perchlorate (trace metal basis) were all purchased from Sigma-Aldrich. The platinum (Pt) sheet counter electrode (99.99% purity, temper as rolled, 0.10 mm thick) was purchased from Advent Research Materials (United Kingdom). Alginate was made with PBS in a 4 : 1 mixing ratio (16 g in 64 mL).

### Synthesis and fabrication

Commercial poly(3,4-ethylenedioxythiophene):poly(styrene sulfonate) (PEDOT:PSS) re-dispersible pellets were first dispersed in DI water to form a 1.1% w/v aqueous solution by stirring in a Duran flask overnight at 150 rpm, room temperature. Aggregation was then induced by adding phosphate buffered saline (PBS) solution to the same flask at 10× concentration in a 90 : 10 volumetric ratio (PEDOT : PSS to PBS), after which the solution was left to stir at the same speed for a further 2 hours. The resulting gels were then treated with acetic acid in volumetric ratios of PEDOT : PSS/acetic acid (1.5 : 1, 1.2 : 1, 1 : 1, 1 : 1.2, and 1 : 1.5). Aqueous suspensions of PEDOT:PSS/acetic acid were added dropwise to a stirring solution of 30 mL light mineral oil containing 2% w/v Span80. The mixture was allowed to stir for 100 minutes followed by the removal of excess solvent *via* centrifugation in 50 mL centrifuge tubes at 2500 rpm for 10 minutes and repeated 3–5 times until the supernatant was clear. Samples were then transferred to 1.5 mL microcentrifuge tubes and centrifuged for a further two cycles at 16 000 × *g* for 5 minutes each.

### Scanning electron microscopy (SEM)

Scanning electron microscopy (SEM) was employed to visualise morphology and structure of the fabricated PEDOT:PSS/acetic acid (PEDOT:PSS/AA) hydrogels (FEI QUANTA 650FEG ESEM, working distance = 23.9 mm, accelerating voltage = 20 kV, spot size = 5.5 nm). Final gel fabrications were dropcast onto clean aluminium stubs and were dried in a vacuum desiccator for one hour. Controls of PBS and PEDOT:PSS + DI water were included. Images were subsequently processed and analysed using Fiji (ImageJ, NIH).

### Injection force characterisation

Mechanical testing was performed using an Instron 5543 (Instron, USA) uniaxial tensile strength machine with a 50 N load cell (Instron, USA) in compressive mode. Samples were mounted in 5/6 mL syringes with an attached 21G Luer lock needle and extruded into a brain tissue phantom. Gel phantoms were prepared by dissolving 0.6% w/v of agarose into boiling PBS. The agarose solution was then transferred to a 1 cm × 1 cm polystyrene cuvette and allowed to cool to room temperature to set. Gels were extruded at 50 mm min^−1^ until a limit of 30 N or 100% breaking point.

### Electrochemical characterisation

Electrochemical measurements were performed using the Multi Autolab M101 potentiostat (Eco Chemie, The Netherlands). Electrochemical impedance spectroscopy (EIS) was performed from 0.1–100 000 Hz using a 0.03 V_RMS_ AC sinusoid. Cyclic voltammetry (CV) was performed with a 0.15 V s^−1^ sweep rate over 11 cycles with a voltage range of −0.6 V to 0.8 V. Electrochemical characterisation was performed using a three-electrode set-up in 0.6% w/v agarose gel, in PBS, with all three electrodes immersed within the gel. To provide a cavity for the conductive gel solution and standardise the electrode size, a platinum (Pt) rod counter electrode with a 1 mm diameter was marked and immersed into the agarose up to this point.

The conductive gel preparation was used as the working electrode (geometric surface area (GSA) = 0.38 cm^2^), along with a Pt sheet counter electrode (GSA = 1.92 cm^2^) and an isolated Ag/AgCl reference electrode. A Kynar-insulated silver 30 awg wire (GSA = 0.11 cm^2^ diameter) was dip-coated in a solution of *N*,*N*′-dimethylacetamide containing PEDOT:PSS and thermoplastic polyurethane (conductive elastomer, CE) solution as sourced from ref. 23 and was immersed in the gel for conductivity measurements. Controls were run with a Pt rod (GSA = 0.376 cm^2^) working electrode and a Pt sheet counter as well as with the CE-coated wire on its own as the working electrode.

### Voltage transients

Voltage transients for conductive gel preparations were obtained using a two-electrode set up. Charge balanced cathodic first pulses with a current amplitude of 12 mA were delivered using a stimulus generator (MC_Stimulus II v3.4.4 software, TG4002, Multichannel Systems, Germany) with a pulse width of 100 μs, an interphase gap of 20 μs and interpulse gap of 20 ms. Resultant voltage transients were visualised using a Tektronix TBS2000 Series Digital Oscilloscope (Tektronix, USA).

### Cytotoxicity

Rat Schwann cells (SCL 4.1/F7) were cultured in serum supplemented cell culture medium (DMEM + 10% FBS + 1% P/S) and routinely passaged at 70–80% confluency in T75 flasks between P6-P12. Cytotoxicity was assessed using the live/dead assay, performed according to the manufacturer's protocol (calcein-AM/ethidium homodimer-1, invitrogen). Schwann cells were seeded in 24-well plates (Corning, USA) with a seeding density of 2.1 × 10^5^ cell cm^−2^ and allowed to attach for 24 hours. Gels of each formulation at a volume of 20 μL were added as a suspension to cell medium in direct contact with the cells. All wells were filled with 1 mL medium. Positive cytotoxicity controls were prepared by diluting 4% v/v, 5% v/v and 7.5% v/v of ethanol into serum supplemented cell culture medium. After staining, the cells were imaged with a Leica SP8 Inverted Confocal Microscope (400 Hz, 512 × 512). Three images were taken of each well from the left, centre, and right regions of the well plate at 10× magnification.

### *Ex vivo* electrochemical characterisation

Adult female Sprague Dawley rats with a weight range of 200–230 g (Charles River Laboratories, United Kingdom) were sacrificed under anaesthesia. All animals were housed and handled in accordance with the United Kingdom Animal (Scientific Procedures) Act 1986 under project licence PA98EF5E9 and approved by institutional ethical review committee (ICL Animal Welfare and Ethical Review Board). The brain and biceps femoris were harvested and kept in ice cold PBS until use. For EIS and CV measurements, a stereotactic frame was first set-up to achieve better control of gel deployment. The brain was placed in an 80 mm glass Petri-dish and filled with alginate/PBS solution (as described previously) to stabilise and prevent movement during handling. A thin layer of PBS was then added on top of the alginate to serve as an electrolyte. For the counter-electrode, a Pt sheet of GSA = 6.431 cm^2^ was used and placed contralaterally to the area of gel implantation, while still in contact with the brain. An Ag/AgCl reference electrode was also placed in contact with the brain, close to the point of implantation. For the working electrode, a stainless-steel needle (19G) was first inserted into the tissue *via* stereotactic frame, through which an insulated CE-coated silver wire (0.3 mm diameter) was threaded through. The 1.5 : 1 gel fabrication was then deployed by attaching a 1 mL syringe to the needle and extruding 50 μL directly into the brain. The needle was then carefully removed to keep the wire in place within the gel. Measurements were recorded for the wire and needle alone without the gel, for the gel with both the wire and needle, and finally for the wire and gel with the needle removed.

### X-ray computed tomography (CT)

Tissue was submerged in Lugol staining solution and maintained for 24 hours. To visualise the injection site and assess the compactness of the gel in tissue, μCT images were acquired (Xradia 510 Versa, Zeiss, GER, 0.4× objective, 80 kV, 7A, 6s exposure) for both the brain and muscle. Images from the μCT were analysed and segmented in IMARIS 10.0 software.

### Statistical analysis

Python Jupyter Notebook was used for all data analysis and visualisation. ANOVA and Tukey's HSD tests were run to check for statistical significance.

## Results

**Fig. 1:**
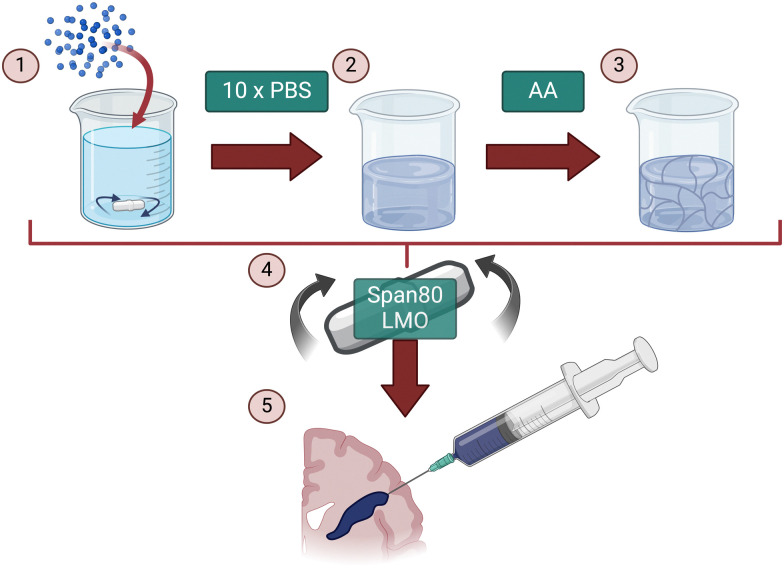
Schematic showing the production process for PEDOT:PSS/Acetic acid gels. (1) PEDOT:PSS redispersible pellets were dispersed in DI water. Solution was left to stir at room temperature overnight at 150 rpm. (2) 10× PBS was added and left to stir for a further 2 hours. (3) Acetic acid was added in varying ratios of PEDOT : PSS/AA (1.5 : 1, 1.2 : 1, 1 : 1, 1 : 1.2 and 1 : 1.5). (4) PEDOT:PSS/AA solutions were transferred to a syringe and extruded dropwise into a Span80 + light mineral oil (LMO) emulsion. (5) Gels were transferred to a syringe for facile administration during material characterisation. [Created with BioRender.com].

**Fig. 2:**
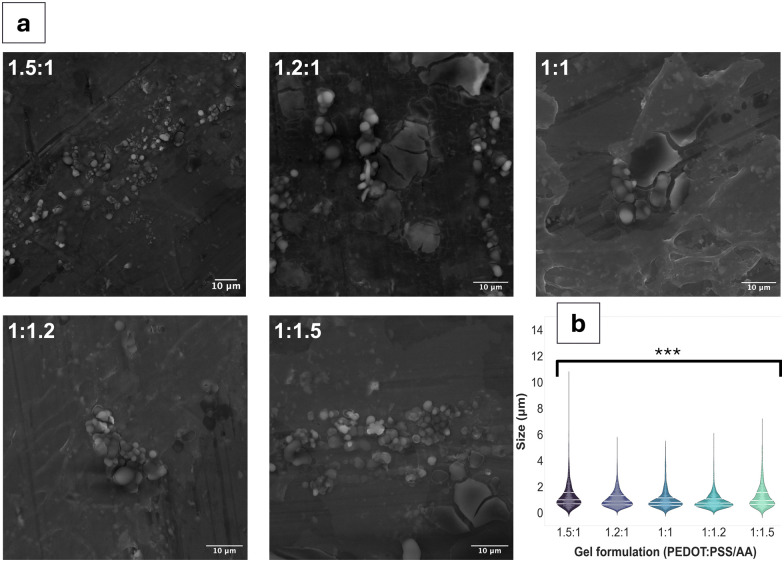
SEM characterisation (a) SEM images of PEDOT:PSS/AA gels for each acetic acid ratio (indicated by PEDOT:PSS/AA ratio in upper left corner). Scale bars for all images = 10 μm. (b) Combined violin-swarm plot showing particle size distribution in gels for each acetic acid ratio (*n* = 1000). Lines within each violin plot mark the median and interquartile range. ANOVA ***p < 0.001 sig. between all groups.

**Fig. 3:**
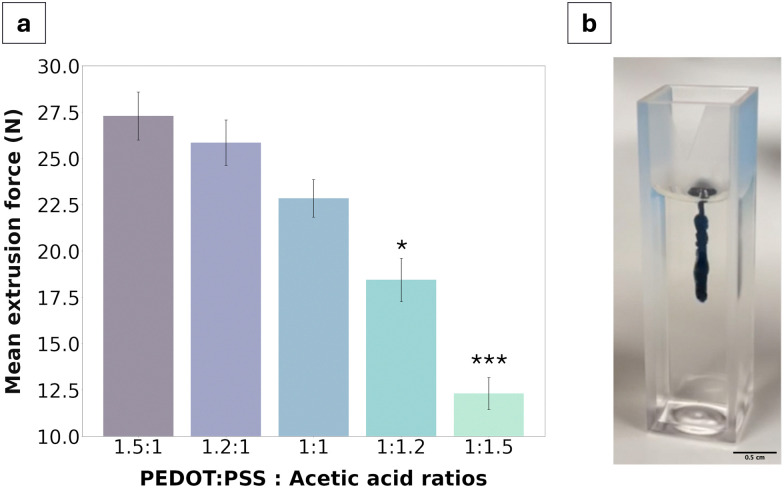
Injectability characterisation of PEDOT:PSS/AA gels. (a) Left-hand panel shows mean extrusion force values for PEDOT:PSS/AA gels when injected into agarose (*n* = 3). ANOVA *p < 0.05, ***p < 0.001 sig. between 1 : 1.2 and 1 : 1.5 groups. (b) Photograph of gel after injection into agarose (PEDOT : PSS/AA ratio used was 1.5 : 1).

**Fig. 4:**
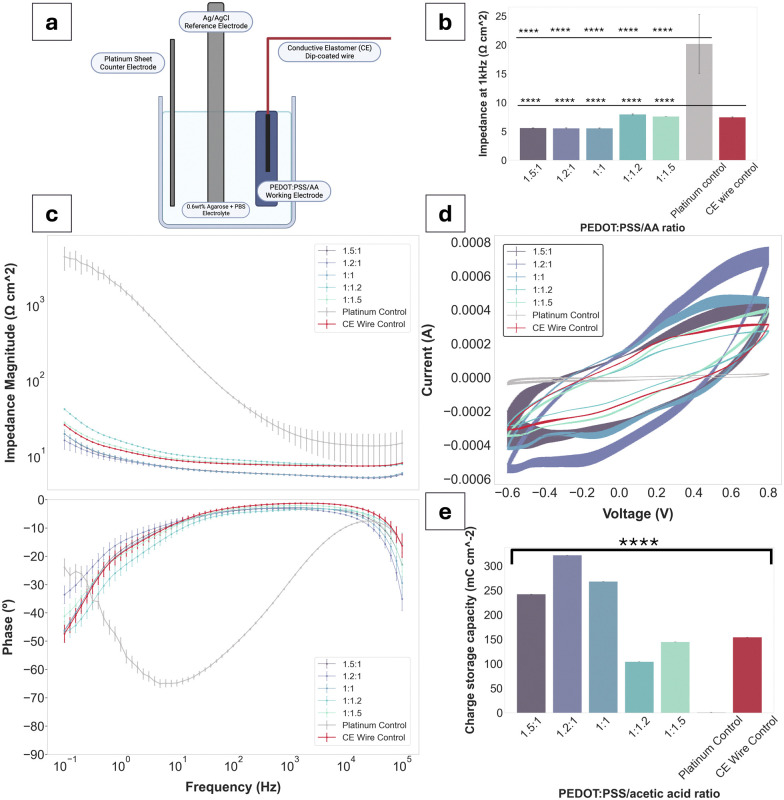
Electrochemical impedance spectroscopy (EIS) and cyclic voltammetry (CV) for PEDOT:PSS/AA gels with Pt and CE wire controls. (a) Schematic of a three electrode set-up for electrochemical characterisation. (b) Impedance magnitude at 1 kHz for gels and controls (*n* = 3). ANOVA, ****p < 0.0001, sig. between all samples compared to Pt control and CE control. (c) Bode plot showing impedance magnitude data for PEDOT:PSS/AA gels and controls (upper panel) and phase angle data for PEDOT:PSS/AA gels and controls (lower panel) (*n* = 3) (*n* = 3). (d) CV data for PEDOT:PSS/AA gels and controls (*n* = 3). (e) Charge storage capacity (CSC) values calculated for gels and controls. ANOVA, ****p < 0.0001, sig. between all groups.

**Fig. 5:**
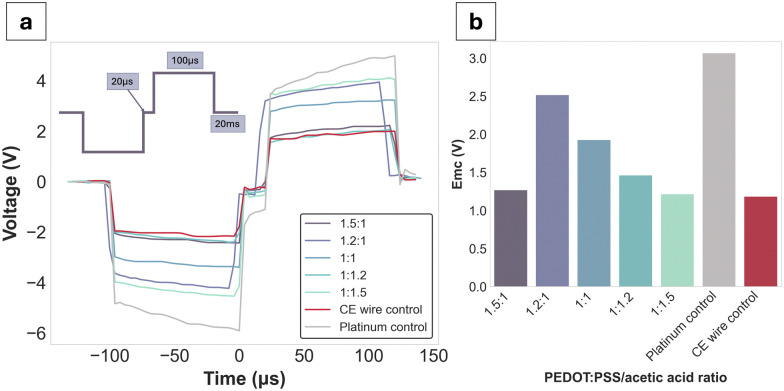
Voltage transients at 12 mA for PEDOT:PSS/AA gels. (a) Voltage transients recorded at 12 mA for PEDOT:PSS/AA gels as well as Pt and CE wire controls. Inset: Biphasic current amplitude applied for recordings was 12 mA, with 100 μs pulse width, 20 μs interphase gap, and 50 Hz (20 ms) interpulse gap. For all measurements, the same agarose-contained set-up was used as previously described in the electrochemical characterisations (*n* = 1). (b) *E*_mc_ values of gels and controls (*n* = 1).

**Fig. 6:**
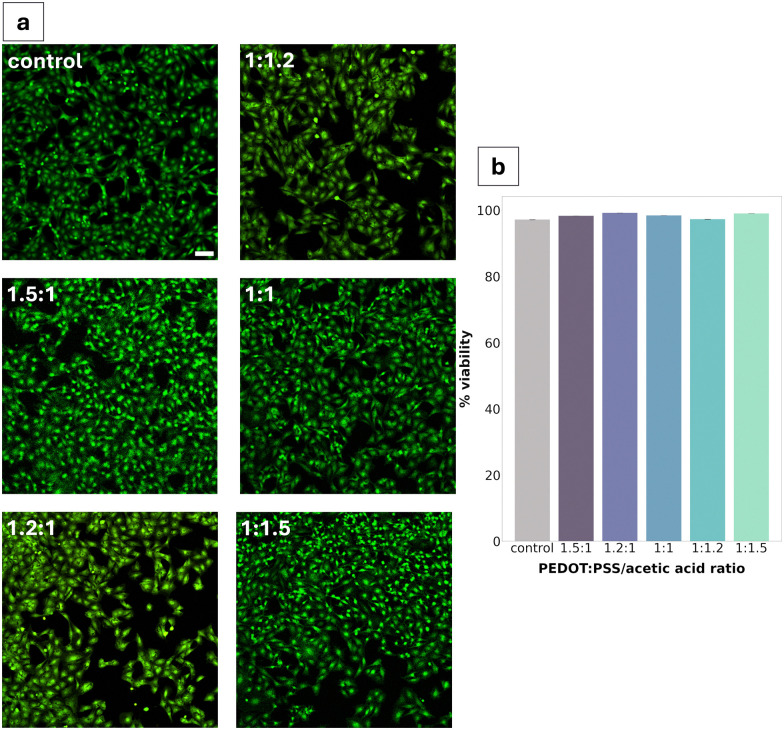
Cytotoxicity of PEDOT:PSS/AA gels in direct contact with Schwann cells. (a) Confocal microscopy pictures of live/dead (calcein-AM/ethidium homodimer-1 stains) assay results for PEDOT:PSS/AA gels in direct contact with cells along with negative TCPS control. Scale bar = 100 μm. (b) Viability percentages of live/dead assay results (*n* = 3).

**Fig. 7:**
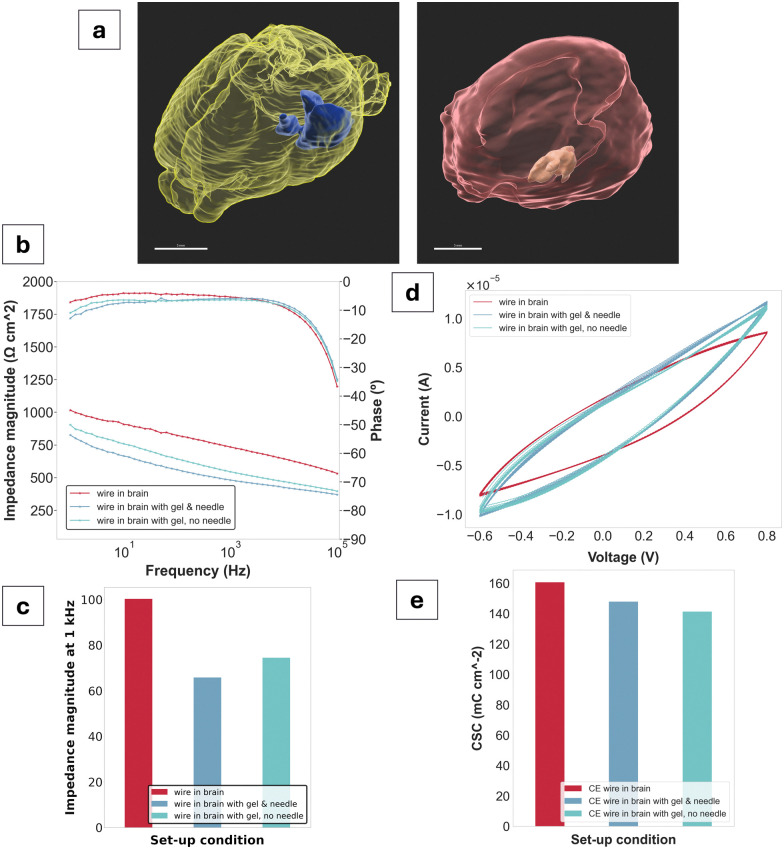
*Ex vivo* characterisation. (a) Reconstructed images of brain (left) and muscle (right) showing gel injection in blue and beige, respectively. Scale bar = 5 mm. (b) Bode plot showing EIS data for CE wire in brain control and two test conditions (*n* = 1). (c) Impedance magnitudes reported at 1 kHz (*n* = 1). (d) CV data (*n* = 1). (e) CSC values (*n* = 1).

The aim of this study was to fabricate an injectable conductive hydrogel system through physical aggregation and oil-based emulsion (Fig. 1). The PEDOT:PSS/AA hydrogel system was characterised at different PEDOT : PSS/acetic acid ratios (1.5 : 1, 1.2 : 1, 1 : 1, 1 : 1.2, and 1 : 1.5) by assessing a series of material properties including microstructure (Fig. 2), injectability (Fig. 3), electrochemical behaviour (Fig. 4 and 5), cytotoxicity (Fig. 6), as well as functional proof-of-concept in *ex vivo* tissue (Fig. 7).

All ratios of the PEDOT:PSS/AA gels produced hydrogels that were mechanically stable and thus suitable for further characterisation. Gel consistency varied based on acetic acid concentration, though all gels were transferable between containers and could be loaded into syringes for use as an injectable system. Material yield following emulsion was marginally higher for gels with higher PEDOT:PSS content (1.5 : 1, 1.2 : 1 and 1 : 1) than for their counterparts.

Qualitative observations derived from the SEM images (Fig. 1a and Fig. S1, ESI†) suggest the coexistence of fibrous and particulate structures across all PEDOT:PSS/AA ratios. However, a notable exception is observed in the fabrication with the highest acetic acid ratio (1 : 1.5), where fibrous structures are absent. The mean particle sizes for all the fabrications were similar, remaining in a narrow range of values. The largest particle sizes, by a small margin were observed for the 1.5 : 1 formulation, with a mean of 1.2 ± 1.0 μm. For the remaining formulations, particle sizes ranged from 1.2 ± 0.9 μm for 1 : 1.5 to 0.9 ± 0.6 μm for 1 : 1.2. Fibre sizes also differed based on the PEDOT:PSS/AA formulation, with the longest fibres on average (62 ± 61 μm) recorded for 1 : 1 while the shortest fibres overall at 43 ± 34 μm were observed for 1 : 1.2. In terms of their surface area, the 1.5 : 1 fabrication registered the highest mean at 3.4 × 10^3^ ± 1.8 × 10^4^ μm^2^ while the lowest surface area at 5.6 × 10^2^ ± 1 × 10^3^ μm^2^ was measured for 1.2 : 1.

The injectability of the material was characterised *via* extrusion force measurements using a tensile strength machine in compressive mode with the goal of quantifying the force required to extrude the gel through a 21G needle. Globally, all extrusion forces measured were below 30 N, ranging from 27.3 ± 0.6 N for the 1.5 : 1 fabrication to 11.1 ± 0.8 N for the 1 : 1.5 fabrication. A clear trend was further identified wherein extrusion force decreased with an increase of acetic acid in the formulation.

Bode plots have been used to display the electrochemical performance of PEDOT:PSS/AA gels, as seen in Fig. 4. Compared to the Pt control, all gel fabrications exhibit a decrease in impedance of approximately two orders of magnitude. Highest impedance magnitudes are recorded for 1 : 1.2 and 1 : 1.5 ratios, while the lowest are exhibited by 1 : 1, followed by 1.5 : 1 and 1.2 : 1. Across the whole frequency spectrum, each gel fabrication experiences a steady decrease in impedance magnitude, with the three formulations with the highest PEDOT:PSS content (compared to acetic acid) at the lower margin. Thus, there is a tentative trend of decrease in impedance magnitude with an increase in PEDOT:PSS content in the PEDOT : PSS/AA ratio. Additionally, the impedance magnitude of the Pt control exceeds both the CE wire control and the gel formulations at all frequencies. The impedance magnitude profile of all gels displays a similar shape to that of the control CE wire, confirming the retention of the electrochemical behaviour of PEDOT:PSS and its charge transfer characteristics. At the biologically relevant frequency of 1 kHz, all gels exhibit impedance magnitudes significantly lower than Pt. Phase angles of all samples are close to 0° and overlap with the curve of the control CE wire, indicative of the pseudocapacitive behaviour characteristic of CPs.

CV and CSC data mirrored the electrical behaviour of the samples seen for EIS, further confirming the pseudocapacitive behaviour displayed by CPs. All fabrications as well as the CE wire control span a larger range of current amplitudes compared to the Pt (−3.8 × 10^−5^ to 2.1 × 10^−5^). Specifically, for the 1.2 : 1 ratio, which recorded the highest peaks, the current amplitude range is from −5.4 × 10^−4^ to 7.1 × 10^−4^. The CSCs of all gel fabrications are much higher than Pt (1.08 mC cm^−2^), with three of the fabrications (1.5 : 1, 1.2 : 1, 1 : 1) exceeding the CSC of the CE wire control. Only the 1 : 1.2 and 1 : 1.5 fabrications have a lower CSC compared to the CE control of 104.2 mC cm^−2^ and 144.2 mC cm^−2^ compared to 154.1 mC cm^−2^, respectively. All other sample values were situated between the Pt as the lower and the CE-coated wire as the higher thresholds.

Voltage excursions were performed in order to assess the charge injection limits (CILs) of the gel formulations alongside the Pt and CE wire controls (Fig. 5a). Since the CILs could not be reached except for Pt (at −3.1 V), the decision was made to obtain stimulus response data at a fixed current of 12 mA, which was close to the equipment limit. Globally, all gel formulations had lower voltage drops compared to Pt. *E*_mc_ values were reported for each of the formulations at the applied waveform, with no clear trend directly attributable to the PEDOT:PSS/AA ratio. The CE wire control had an *E*_mc_ value of −1.1 V, which was closest to the PEDOT:PSS/AA fabrication with the highest acetic acid content (1 : 1.5) at −1.2 V.

Cytotoxicity was assessed *via* live/dead assay after 24 h of direct exposure of the material to the cells. All PEDOT:PSS/AA gel fabrications were found to be non-cytotoxic, with the highest viability (99.2%) seen for the 1.2 : 1 ratio (Fig. 6b). Compared to the TCPS control, no significant differences were observed in cell morphology. Cell density appeared to be decreased in two of the fabrications (1.2 : 1 and 1 : 1.2).

To assess the viability of the gel as a tissue-injectable construct, electrochemical impedance spectroscopy (EIS) and cyclic voltammetry (CV) were performed *ex vivo* using a three-electrode set-up in a rodent brain. Overall, a decrease in impedance was observed for the gel compared to a CE-coated wire control. At the biologically relevant frequency of 1 kHz, a decrease of approximately 200 Ω was measured between the CE-coated wire (730 Ω) and the gel in contact with the wire (540 Ω). Phase angles showed PEDOT-like behaviour,^24^ exhibiting a tendency towards zero at low frequencies. CV waveforms also showed pseudocapacitive behaviour characteristic of PEDOT.^23^ The CSC values are all in a range from 141 mC cm^−2^ for the CE wire interfacing with the gel alone to 161 mC cm^−2^ for the CE wire control. A decrease in CSC is therefore observed upon gel injection compared to the control.

## Discussion

Overall, gels were successfully produced for all PEDOT:PSS/AA formulations, and were characterised as injectable, conductive, non-cytotoxic, and capable of safe charge injection. The mechanical and electrochemical properties of the manufactured gel system were also independently tuneable, with particle sizes in a single-micron range. Furthermore, *ex vivo* proof-of-concept demonstrated the material as stable and compact when injected into both brain and muscle tissue. Assessment of the gel's EIS and CV whilst injected yielded a decrease in impedance compared to a CE-coated wire control, which was indicative of favourable electrochemical properties that are retained both in an *in vitro* and *ex vivo* context. A major outcome of the study was demonstrating that a facile method of fabrication relying on commercially available, non-toxic components, is still able to produce a highly translatable material.

The fabrication of many injectable hydrogel systems often entails highly intricate and costly manufacturing processes, primarily due to their reliance on specialised equipment such as UV for photo-crosslinking, and meticulous control of conditions like temperature and pH.^25^ Moreover, numerous systems with prospective applications for tissue interfacing within the human body are fabricated from ECM-derived components like collagen^26^ or hyaluronic acid,^27^ which tend to be expensive due to the complex extraction and purification routes involved.^28^ A major advantage of the present material thus also lies in its relatively facile and inexpensive formulation. Namely, the entire process is performed at room temperature with commercially readily available, non-toxic materials, with no requirements for specialised conditions like temperature or pressure controls. Consequently, the material exhibits stability and can be stored under standard conditions, facilitating broad applicability and translation into a range of practical contexts.

An important facet of the PEDOT:PSS/AA gels dictating their viability for neural interfacing emerges from their reliance on physical cross-linking, as opposed to chemical. Herein, the addition of concentrated PBS was the primary facilitator of this process by inducing aggregation of the PEDOT:PSS colloidal dispersion. After the acetic acid rearranged these aggregates to recover electrical conductivity, batch emulsion was then necessary to restore the required consistency of the material for further use as an injectable system. As a result, each of these components was essential not only in achieving injectability of the material, but also for defining the final system as a physically aggregated gel. Furthermore, as an injectable, physically cross-linked system, it is not limited by a need to follow a specific pattern of arrangement when implanted, thus rendering it less prone to structural disturbance by micromovements of the surrounding tissue. This flexibility in placement and anatomical adaptability makes the present material particularly suitable for neural interface applications, where precise implantation is crucial for successful integration and function. The material would also benefit from relying on a less complex delivery method by allowing for minimally invasive injection into neural tissue, which is surgically feasible.

A major objective for this study focused on investigating the influence of acetic acid for tuning the material's properties. As a rationale for choosing acetic acid for synthesis herein, guidance was drawn from literature reports of treatment with acids like sulfuric acid^20,21^ being used to recover electrical functionality and improve gel conductivities.^22^ Interestingly, however, while the acetic acid was initially chosen as a means of recovering electrical functionality, the formulations with higher PEDOT:PSS content consistently outperformed their higher acetic acid counterparts during electrochemical characterisation. A possible explanation for this phenomenon emerges from acetic acid acting as a polar solvent when combined with PEDOT:PSS, which may facilitate swelling while in solution.^22^ Indeed, during fabrication, the effect of acetic acid in solution was immediately apparent, with a lower viscosity observed as the acetic acid content was increased. Subsequently, this complicated handling of the 1 : 1.2 and 1 : 1.5 gel formulations during fabrication due to their lower packing, resulting in difficulties with separating from the supernatant and an overall lower yield. As a result, clear conclusions could be drawn about the importance of the material's structural integrity following aggregation wherein an excess of acetic acid has a disruptive effect. An extension to exploring acetic acid-induced gel modification was also to investigate whether an optimal PEDOT : PSS/AA ratio could be determined. Thus, considering their performance across all material characterisation tests, the gels with a higher PEDOT:PSS content (particularly 1.5 : 1 and 1.2 : 1) were selected as optimal, with the 1.5 : 1 later being chosen for *ex vivo* testing.

To further tune gel properties by modifying the particle size, batch emulsion was employed for the fabrication. Morphologically, both fibrous and particulate structures were evident, resulting from micelle-assisted formation.^29^ Overall, qualitative observations clearly pointed towards changes in the gel's viscosity and compactness following oil emulsion. Recently, a micelle-templated chemical oxidative polymerisation of PEDOT:PSS was proposed where researchers investigated the effects of different surfactants on stability, conductivity, and other properties, finding significant variations in critical micelle concentration (CMC) and thermal stability.^30^ The morphology and size of the particles produced were also dependent on the surfactant employed, with largest particles being produced by the non-ionic surfactant Triton X-100 at 56.87 ± 5.54 nm, while at 2.5 CMC.^30^ In the present study, batch emulsion by Span80, which is also a non-ionic surfactant, produced particles in a range approximately twice as large in comparison. Further research could focus on investigating the CMC as applied to this fabrication process, thus finding a balance between ease of fabrication and precise property control during manufacturing.

Quantifying injectability of the PEDOT:PSS/AA gels was of particular importance for assessing the clinical applicability of the material. Namely, to be clinically viable, injection into human tissue cannot safely exceed 30 N, which is the highest force that can be comfortably exerted in a hand-held syringe grip.^14,31,32^ Therefore, since the results showed that the highest polymer-to-acetic acid ratio (1.5 : 1) reached a force of 27.3 ± 0.6 N, formulations with a higher relative volume of PEDOT:PSS were deemed unviable and were not tested. The resulting values from the injection force test described the pressure applied for continuous outflow and ranged from 100 to 222 kPa, from the 1.5 : 1 to the 1 : 1.5 ratios, respectively. When compared to the range of values clinically measured in equivalent 5 mL syringes for injection into tissue lesions, the pressures measured in this study were much lower, with a five-fold decrease from a threshold of approximately 500 kPa.^33^ From this perspective, the results of the extrusion force tests are thus promising for all gel fabrications. The lowest extrusion forces were observed for gels with the highest acetic acid content and exhibited lower solution viscosity. Notably, aqueous dispersions of PEDOT:PSS have been reported to exhibit shear thinning behaviour such that the solution behaves as a viscoelastic solid once inside the body.^34,35^ Deep eutectic solvents which can be produced from organic acids such as acetic acid offer a route to improve local material stability by supporting molecular interactions underpinning gelation and have been demonstrated to improve mixed charge transport of PEDOT:PSS.^36^ Moreover, locally available biologically relevant molecules such as choline or amino acids can be used to support local assembly of reinforced PEDOT:PSS/acetic acid gels *in situ* as part of a biosynergistic approach to electrode fabrication.^36^

Investigating its electrochemical behaviour has formed the backbone of the material's characterisation tests to best evaluate its potential as an electrode. The set-up for measuring the gel's EIS and CV was specifically designed to assess its electrochemical properties within a context as similar to its target environment as possible, while focusing on how these properties are informed by its internal structure. As a physically-aggregated system, gelation of the PEDOT:PSS/AA gels was achieved by overcoming the dispersion of PEDOT:PSS chains through macromolecular physical entanglement. This contrasts with strategies of achieving conductivity through the involvement of interpenetrating polymer networks (IPNs) or dual networks (DNs), which typically rely on chemically cross-linked structures of polymer networks.^37^ IPNs and DNs are advantageous for electrical conductivity due to their enhanced mechanical stability and increased cross-linking density, resulting in a denser and more interconnected structure that facilitates electron transport.^38^ Their composition and structure can also be precisely controlled, allowing for tuneable electrical properties. Yet, while these electrochemical properties render them well-suited for neural electrodes, biosensors, and other bioelectronic devices, they are not conducive to injectability since they form bulk gels that fragment upon shear force without re-gelling. Moreover, previous findings have posited that structural arrangements of individual particles are in fact conducive to enhanced conductivity due to their increased surface area.^13^ Thus, the present study has proposed an injectable system whose reliance on physical aggregation is not at the detriment of its conductive properties.

To further electrochemically characterise the PEDOT:PSS/AA material, the gel was also stimulated while in the same set-up as previously described. Overall, even though the current applied was close to the equipment limits at 12 mA, it was still not nearly sufficient to reach the CIL of the gels, so only *E*_mc_ values could be reported. This observation is crucial as it suggests that the electrode material can efficiently deliver and accept high levels of charge without excessive accumulation, which is essential for an electrode's safe and effective stimulation. Moreover, this ability to be safely injected with large amounts of current is a critical consideration for the design of implantable electrodes to avoid reaching the water electrolysis window, which would result in irreversible reactions detrimental both to the electrode's functionality and the surrounding tissue.^39^

For the stimulation paradigm, a biphasic current was chosen because it allows for a balanced charge injection and extraction, minimising electrode polarisation. *In situ*, this would mitigate the risk of tissue damage while facilitating precise control over neural stimulation parameters. The pulse width of 100 μs was selected since it has been reported as well within the bounds for a range of neural applications including stimulation of the optic nerve (25–400 μs), and DBS (60–200).^40^ However, there is a narrow window of safety and applicability in terms of the size of implantable electrodes in the brain. For DBS, electrodes are considered relatively large and can reach up to a GSA of 0.06 cm^2^ (ref. 41) while penetrating microelectrodes have GSAs around 2000 μm^2^.^42^ For reference, the surface area of common DBS targets for Parkinson's Disease, for instance, is 0.158 cm^2^ for the subthalamic nucleus (STN) and 0.478 cm^2^ for the globus pallidus interna (GPi).^43^ Thus, considering the efficient charge transfer characteristics of the gel electrode, future work in down-scaling to adapt to the specific anatomical requirements would be highly feasible.

Cytotoxicity studies reported high viability (>95%) across all gel fabrications, with minimal changes in cell density. The consistency of this high viability across all five formulations can be largely explained through the inherent cytocompatibility of the gel's constituents.^44^ In addition to being a promising result for advancing the characterisation of the PEDOT:PSS/AA gels towards their prospective application in neural electrodes, the non-cytotoxicity of the material is consistent with previous work in literature.^45^ For instance, in a paper studying the cytotoxicity of PEDOT:PSS for peripheral nerve regeneration, Schwann cells were found not to experience any toxic effects after seven days. In the present study, Schwann cells were selected as the model cell system primarily due to their high sensitivity to changes in their microenvironment and since they have been well-documented for use in cytotoxicity studies. Future studies could investigate differences in the cytotoxicity of the material for other cell lines.

Venturing more broadly beyond *in vitro* studies, it is important to consider the cellular effects and interactions of the material when implanted long-term into the tissue environment. Namely, PEDOT:PSS thin films have been shown to erode during long-term use in wet environments.^46^ Coupled with this are reports of PEDOT additives leaching when in a swollen state. Specifically, PEDOT:PSS is fabricated with the PSS chains in excess to promote the aqueous dispersion of the complex. As a result, the excess PSS can leach out from the PEDOT:PSS, which is toxic for cells due to its low pH.^23,47^ Thus, considering approaches for overcoming this potential concern would be vital when assessing the behaviour of PEDOT:PSS/AA gels in their intended environment. One proposed strategy for overcoming this is by employing chemical stabilisers like 3-glycidoxypropyltrimethoxysilane (GOPS) to lock the PEDOT:PSS chains and prevent re-dispersion, thereby improving the physical stability, as shown by Pires *et al.*^46^ Here, researches also showed that cross-linked PEDOT:PSS was non-cytotoxic to neural stem cells. Alongside proposing a method of circumventing the issues associated with PSS toxicity *in situ*, cross-linkers like GOPS might pose an interesting avenue for investigating their effect on the material's injectability. A similar strategy has been employed by cross-linking PEDOT:PSS with poly(*N*-isopropylacrylamide) (PNIPAM) wherein the self-healing behaviour of the gel was significantly improved, whilst simultaneously heightening the material's conductivity.^48^

As an injectable system, a potential consideration is the ability to incorporate cells into the material itself, thereby assessing the possibility for non-cytotoxic encapsulation. While the PEDOT:PSS/AA gels have exhibited no cytotoxicity in direct contact with the cells used herein, encapsulation studies would progress this towards a more holistic understanding of the cytocompatibility. To achieve cell encapsulation, a crucial element missing from the current system is the addition of bioactive motifs, such as for cellular adhesion, anti-inflammation, and in support of proliferation. Biofunctionalization of PEDOT:PSS has been extensively explored^49–51^ for its application in tissue scaffolds. One such study used polylysine, heparin, basic fibroblast growth factor and fibronectin to modify PEDOT microfibres, finding that the added epitopes promoted tissue healing following spinal cord injury.^52^ Similarly, peptides like YIGSR and IKVAV, both laminin-derived adhesion motifs, have been shown to improve adhesion and differentiation of neural cells when added to PEDOT.^51,53^ Taken together with the focus on injectability, an interesting avenue would therefore be to explore the potential for injecting a material with cells already encapsulated. Previous such work has centred on cell delivery with alginate-based hydrogels.^54^ Incorporating cells in this way would be predicted to enhance the acceptance of the gel *in situ* by facilitating seamless integration with the surrounding tissue.

The *ex vivo* studies were highly pertinent to classifying the PEDOT:PSS/AA gel system as a viable neural electrode. In addition to proving its injectability in tissue, as well as its compactness and stability, the study yielded initial results displaying its electrical functionality in this context. The decrease in impedance magnitude upon gel injection compared to the control, as well as the overall CP-like behaviour shown by the phase angles and CV as further validation. For the purposes of this study, the gel was deployed *via* a needle held by a stereotactic frame, with the needle acting as a trocar to achieve contact between the gel and the threaded wire. The realisation of this set-up indicates that a minimally invasive implantation approach is highly feasible, further supporting the material's target application.

μCT images taken post-electrochemical characterisation show distinct compartmentalisation of the gel within the tissue without diffusion into the brain's ventricular system. The gel was also injected into muscle tissue to gain a better understanding of whether there was any observable tissue-specificity for its injection. Upon volume reconstruction, no major loss of material after initial injection was observed for either tissue, affirming its structural fidelity. To compare, 50 μL of material was injected into the brain while 20 μL was injected into the muscle, with values of 46.4 μL and 16.6 μL obtained for the reconstructed volumes, respectively. Furthermore, no breakdown or particle dissolution of the gel was observed following *ex vivo* injection, even after staining with iodine (Lugol). While this study was focused on preliminary experimentation *ex vivo*, for a similar PEDOT:PSS and acetic acid-based hydrogel system developed by the Bao group,^12^ injections of 4 μL total were made into the brain tissue of rats *in vivo* after mixing with a cellulosic binding polymer (hydroxymethylpropylcellulose, HPMC). Researchers observed no significant inflammatory response to the gel compared to a needle control and determined the gels remained compact within tissue, even after slicing and staining for histology. In terms of further assessing the thermal and mechanical stability *in situ*, PEDOT:PSS has also been shown as a facilitator in both regards, while maintaining the necessary electrochemical properties.^55^ These findings not only support the presently observed stability of the gel in tissue, but are also a promising motivation for further material characterisations in both *ex vivo* and eventually *in vivo* tissue.

Overall, the PEDOT:PSS/AA hydrogel presents an easily-fabricated and versatile system whose properties are tuneable to generate a compliant electrode interface. In addition to its potential as a neural interface, its cytocompatibility and electrochemical properties position it just as well for further applications as an injectable tissue scaffold. Simultaneously, its physically cross-linked structure and high conformability render it an ideal candidate for use as a bio-ink in 3D printing.

The gel's charge properties and stability in tissue also support its potential as a stable chronic interface upon implantation. Specifically, a high CSC is favourable for a chronic interface because it allows for the delivery of larger charge pulses, which may be necessary for sustained neural stimulation or modulation over extended periods. Moreover, the material's ability to withstand high stimulation pulses without reaching the CIL at the water window is a good indicator of its favourable charge transfer characteristics and safety for use post-implantation. Further studies would therefore need to investigate biodegradability as well as self-healing behaviour, both of which would be quintessential aspects of manufacturing a long-term implant. Investigations of biodegradability and reversibility could also promote application as a transient interface, linking to paradigms for drug delivery wherein the delivery vehicle is set to degrade after use.^56^ For either application, future work should also focus on assessing the potential for infection and consequent risk of failure. Given that the system is designed as minimally invasive, these anticipated risks would likely be reduced compared to its invasively implanted counterparts.

A major future consideration for this material would be determining how best to interface with and power it following injection. One proposition might emulate the set-up currently used for DBS where a lead is threaded from the electrode to a subcutaneously-implanted pulse generator, usually in the clavicle.^57^ This approach would have the benefit of being applied to a surgical procedure that has become largely routine and would also be more closely translatable based on the existing *ex vivo* characterisation set-up (CE-coated wire interface). However, even though it is a standard procedure, DBS leads have been shown as prone to fracturing and displacement.^58^ Similarly, the pulse generator has been associated with complications like infection arising from internal repositioning, or an increased frequency of surgeries to replace the batteries.^59^ Thus, an innovative avenue to explore would be in the sphere of wireless interfacing, such as with the novel technique of temporal interference (TI) stimulation. TI involves administering multiple electric fields with varied frequencies within the kHz range that converge to drive neural activity at a controlled focal point.^60^ Recently, for instance, it has been successfully demonstrated in DBS of the human hippocampus.^61^ Upon injection, the PEDOT:PSS/AA gel could act in concert with TI by serving as a conduit for better-directed electrical stimulation.

## Conclusions

Ultimately, a novel type of injectable PEDOT:PSS hydrogel has been produced, with tuneable mechanical and electrochemical properties informed by acetic acid content. Batch emulsion was also successful in influencing the particle size. Initial material characterisations have demonstrated it as injectable, conductive, and cytocompatible, with a high capacity for safe charge injection. The material further benefits from its facile, easily translatable synthesis. Integration with bioactive epitopes and/or combination with wireless neural stimulation paradigms support new directions for minimally invasive neural interfacing.

## Author contributions

All authors have read and agreed to the final version of the manuscript. I. K. and A. L. wrote the original version of the manuscript, with all authors being involved in review and editing. R. A. G. acquired funding for the study and, along with J. A. G., and A. L., conceptualised and supervised the project. SEM data collection and analysis was performed by I. K. and A. L. Mechanical data was collected and analysed by I. K. EIS CV data was collected by I. K. and analysed by I. K., A. L. and E. A. C. Voltage transients data was collected by A. L. and I. K. who analysed the data along with E. A. C. Cytotoxicity studies were performed by J. K., S. M. A., I. K. and A. L. with data analysis by I. K. *Ex vivo* studies were conceptualised and performed by R. A. G., E. A. C., Z. K. B., A. L. and I. K. E. I. S. and C. V. data were processed by I. K., E. A. C., and A. L., Z. K. B. obtained the rodent tissue for *ex vivo* studies and took the CT images, which were reconstructed and analysed by A. L.

## Data availability

The datasets generated during and/ or analysed during the current study are not publicly available due to ethics and IP but are available from the authors on reasonable request.

## Conflicts of interest

There are no conflicts to declare.

## Supplementary Material

TB-012-D4TB00679H-s001
