# Supplementary material for: Injectable conductive hydrogel electrodes for minimally invasive neural interfaces
Source: J Mater Chem B. 2024 Jul 30;12(36):8929–40. doi: 10.1039/d4tb00679h (PMC11325676; doi:10.1039/d4tb00679h)
Supplement: TB-012-D4TB00679H-s001 [file TB-012-D4TB00679H-s001.pdf]

Supplementary Information (SI) for Journal of Materials Chemistry B.
This journal is © The Royal Society of Chemistry 2024

Supplementary Information

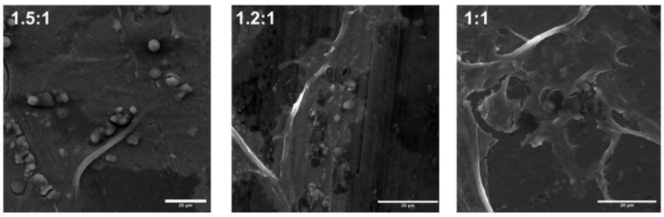

SI Figure 1. *Representative SEM images of fibres for 1.5:1, 1.2:1, and 1:1 formulations.* Scale bars for all images = 20 µm.

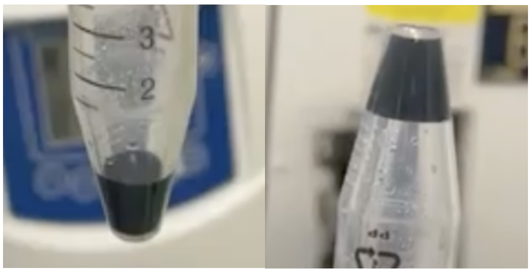

A **B**

SI Figure 2. *Representative images showing final PEDOT:PSS/AA gel.* (A) PEDOT:PSS/Acetic acid gel following batch emulsion and after removing supernatant post-cleaning. (B) When inversed, gel self-supports against gravity. Scale bars for both images = 1 cm.
